# Supplementary material for: Epidermal cell fusion promotes the transition from an embryonic to a larval transcriptome in C. elegans
Source: Development. 2025 Dec 22;152(24):dev205089. doi: 10.1242/dev.205089 (PMC12772960; doi:10.1242/dev.205089)
Supplement: Supplementary information [file develop-152-205089-s1.pdf]

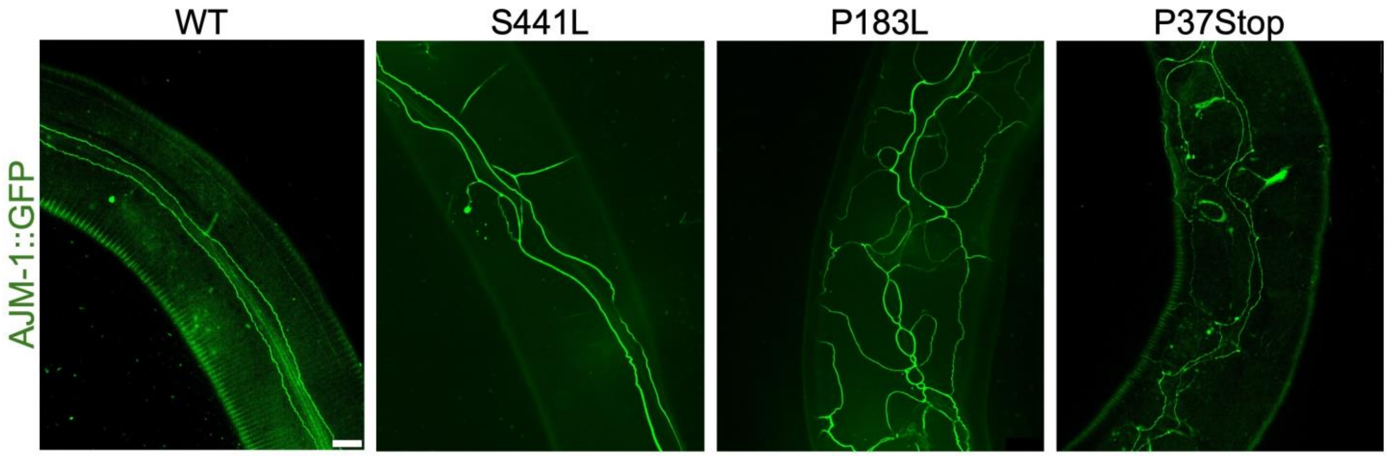

Fig. S1. Fusion defects in *eff-1* mutant adults.

A) Representative images of AJM-1::GFP fluorescence, a marker of apical cell–cell junctions in the epidermis, in N2 (WT) and *eff-1* mutant (S441L, P183L, P37AStop) adults. Scale bar is 10 μm.

Development • Supplementary information

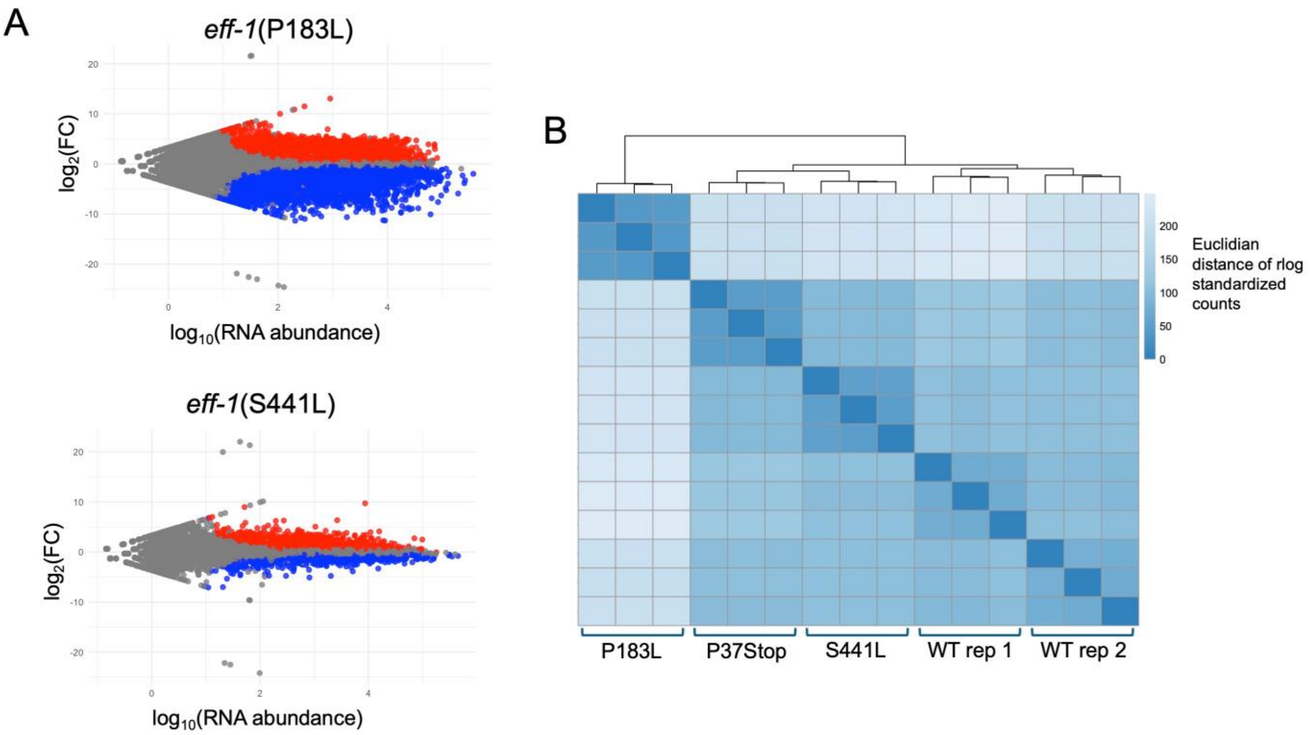

Fig. S2. RNAseq of *eff-1* mutants shows substantial overlap in gene expression changes.

A) MA plots of *eff-1*(P183L) and *eff-1*(S441L) with DEGs (adjusted $p < 0.05$) in red for upregulated and blue for downregulated. B) Euclidian distance matrix of regularized log (rlog)-transformed RNAseq counts of *eff-1* mutants and WT controls from multiple sequencing runs. WT rep 1 and rep 2 refer to the two biological replicates.

Development • Supplementary information

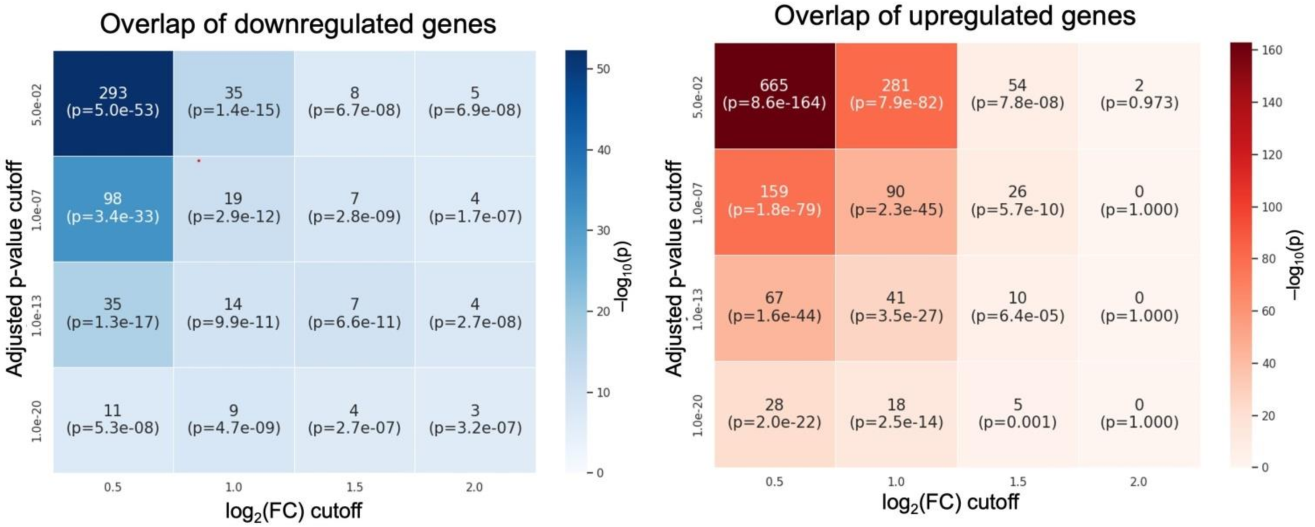

Fig. S3. p-Value and fold-change cutoff analysis of RNAseq results.

Adjusted p-values and $\log_2$(fold change) cutoff analysis for upregulated and downregulated genes. Values are shown for overlap among P37Stop, P183L, and S441L differentially expressed genes. Inset p-values are hypergeometric calculations for significance of the overlap at each set of cutoff parameters.

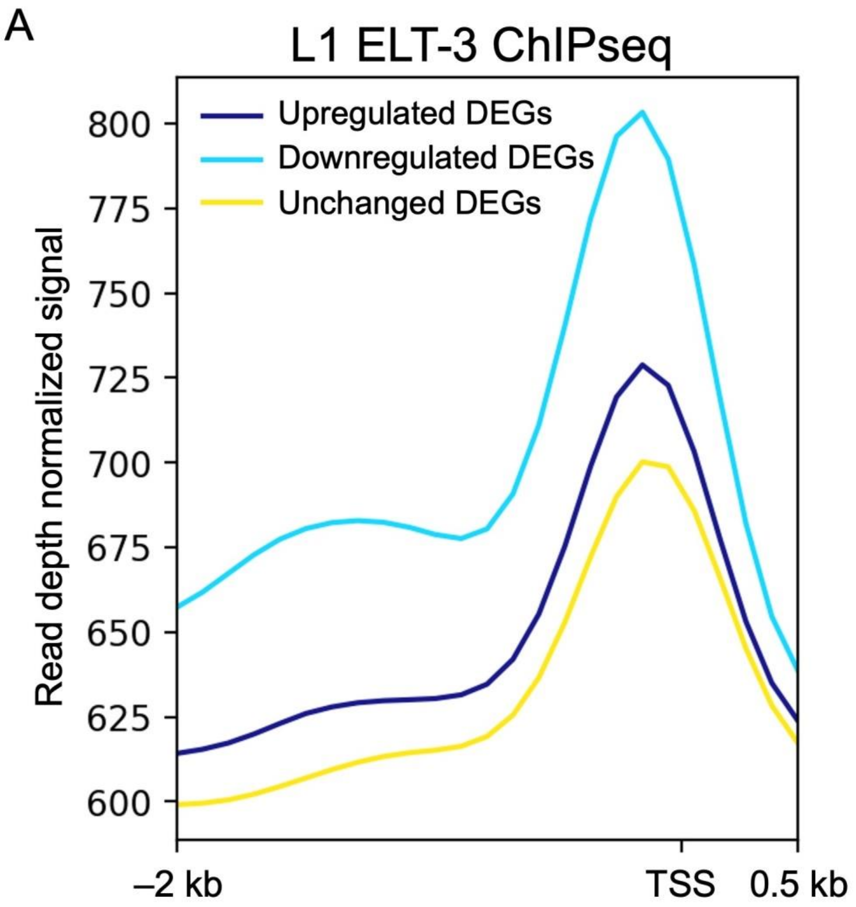

B

	Average $\log_2$ Fold Change	Average Adjusted p-value
**ELT-3 associated down DEGs**	−0.640	0.00454
**Non-ELT-3 associated down DEGs**	−0.494	0.00575
**Statistical Comparison (Kolmogorov–Smirnov test)**	1.492e-08	3.18e-05

Fig. S4. ELT-3 ChIPseq signal across *eff-1* differentially expressed genes.
A) L1 ELT-3 ChIPseq profile across DEGs from *eff-1*(P37Stop). ELT-3 ChIPseq signal was derived from modENCODE data and plotted for a region spanning 2 kb upstream and 500 bp downstream from the transcription start site (TSS) of upregulated, downregulated, and unchanged genes. B) Downregulated DEGs were split into ELT-3–associated and non–associated genes, and adjusted p-values and fold changes were calculated for these two sets. A Kolmogorov-Smirnov test was performed for both distributions.

Table S1. Differential expression analysis

Available for download at
https://journals.biologists.com/dev/article-lookup/doi/10.1242/dev.205089#supplementary-data
